# RhoGDI in RBL-2H3 cells acts as a negative regulator of Rho GTPase signaling to inhibit granule exocytosis

**DOI:** 10.1093/jleuko/qiae150

**Published:** 2024-06-29

**Authors:** Eric L Zhang, Jennifer Van Petten, Gary Eitzen

**Affiliations:** Department of Cell Biology, University of Alberta, Edmonton, Alberta, Canada; Department of Cell Biology, University of Alberta, Edmonton, Alberta, Canada; Department of Cell Biology, University of Alberta, Edmonton, Alberta, Canada

**Keywords:** degranulation, FcεR1, mast cell, Rac1, RhoA

## Abstract

Mast cells are hematopoietic-derived immune cells that possess numerous cytoplasmic granules containing immune mediators such as cytokines and histamine. Antigen stimulation triggers mast cell granule exocytosis, releasing granule contents in a process known as degranulation. We have shown that Rho GTPase signaling is an essential component of granule exocytosis, however, the proteins that regulate Rho GTPases during this process are not well defined. Here we examined the role of Rho guanine-nucleotide dissociation inhibitors (RhoGDIs) in regulating Rho GTPase signaling using RBL-2H3 cells as a mast cell model. We found that RBL-2H3 cells express two RhoGDI isoforms which are primarily localized to the cytosol. Knockdown of RhoGDI1 and RhoGDI2 greatly reduced the levels of all Rho GTPases tested: RhoA, RhoG, Rac1, Rac2, and Cdc42. The reduction in Rho GTPase levels was accompanied by an increase in their membrane-localized fraction and an elevation in the levels of active Rho GTPases. All RhoGDI knockdown strains had altered resting cell morphology, although each strain was activation competent when stimulated. Live cell imaging revealed that the RhoGDI1/2 double knockdown (DKD) strain maintained its activated state for prolonged periods of time compared to the other strains. Only the RhoGDI1/2 DKD strain showed a significant increase in granule exocytosis. Conversely, RhoGDI overexpression in RBL-2H3 cells did not noticeably affect Rho GTPases or degranulation. Based on these results, RhoGDIs act as negative regulators of Rho GTPases during mast cell degranulation, and inhibit exocytosis by sequestering Rho GTPases in the cytosol.

## Teaser

We show RhoGDIs reduce mast cell granule exocytosis via negative regulation of membrane binding and inhibition of activity of Rho GTPases.

## 1. Introduction

Mast cells are tissue-resident leukocytes that reside in epithelial and mucosal tissues throughout the body. A defining mechanism of mast cells is IgE-mediated immunity and FcεRI receptor signaling for proinflammatory processes such as degranulation.^1^ For mast cell degranulation to occur successfully, the cortical F-actin, normally acting as a barrier to granule release, must be rearranged.^2^ Remodeling of the cytoskeleton is seen as peripheral membrane ruffles through F-actin staining, with stimulation of degranulation triggering ruffle formation; lack of membrane ruffling corresponds with inhibition degranulation.^2^ These changes in the actin skeleton in response to stimuli are primarily mediated by the Rho subfamily of small GTPases. Rho GTPases act as signaling molecules in numerous cellular processes involving the cytoskeleton including vesicle trafficking, cell proliferation, spreading, and migration.^3^ GTPases act as bimolecular switches, cycling between their GDP-bound (inactive) and GTP-bound (active) forms via regulation by RhoGEFs (Rho guanine-nucleotide exchange factors), RhoGAPs (Rho GTPase activating proteins), and RhoGDIs (Rho guanine-nucleotide dissociation inhibitors).^4^ We have shown that the addition of Rho GTPase inhibitors suppresses mast cell degranulation which links Rho GTPase regulation to the mast cell activation mechanism.^5^ However, specific targets of Rho GTPase regulation in mast cells have not been well defined, while downstream remodeling of the actin cytoskeleton is consistent with mechanisms required for degranulation in mast cells^5–8^ and exocytosis in general.^9–11^

RhoGDIs are a family of proteins that act as chaperones, regulating the activity and localization of Rho GTPases. RhoGDIs directly interact with Rho GTPases through protein–protein and protein–lipid interactions. The N-terminal domain of RhoGDIs consists of a flexible arm that interacts extensively with the switch I and II regions of Rho GTPases.^12^ RhoGDI binding to the switch regions blocks downstream effector interactions and prevents the release of the currently bound nucleotide. It can also inhibit the GTP hydrolytic activity of the GTPase, preventing regulation by RhoGEFs and RhoGAPs and effectively locking the GTPase in its current state. The C-terminal domain of RhoGDIs contains a geranylgeranyl-binding pocket which does not affect GDP-GTP cycling,^12^ but instead provides for a chaperone-like mechanism that stabilizes Rho GTPases and prevent their degradation by the cytosolic proteasome.^13^ Rho GTPases possess a C-terminal isoprenoid moiety that allow the GTPases to localize to and stably associate with membranes, but the hydrophobicity of the isoprenoid moiety results in instability in the cytosol.^13^ RhoGDIs can extract Rho GTPases from membranes and sequester them in the cytosol,^14^ maintaining a large reservoir that can be utilized at any time.

As RhoGDIs can extract Rho GTPases from membranes,^14^ they may also be responsible for the delivery of GTPases to different target membranes from the cytosol and thus initiate Rho signaling. Rac1, a major Rho GTPase,^3^ is known to drive the formation of lamellipodia and has been observed at the plasma membrane of the leading edge of motile cells.^15^ Another Rho GTPase, Cdc42, drives the formation of filopodia past the leading edge of extending lamellipodia.^16^ A mutant Cdc42 unable to interact with RhoGDI was unable to localize to the plasma membrane, instead accumulating in the perinuclear region.^17^ More recently, RhoGDIs were directly imaged being able to extract inactive and active Rho GTPases both in vitro and in vivo.^18^ Extracted active GTPases may then be shuttled back to the plasma membrane or other membrane compartments without the need to be activated again.

The activity of RhoGDIs results in the tight spatiotemporal regulation of Rho GTPases, and the formation and dissociation of Rho GTPase::RhoGDI complexes is essential to this regulation. Certain lipid microenvironments can influence the release of Rho GTPases from RhoGDIs, with acidic lipids being able to disrupt the complex to allow recruited GEFs to facilitate nucleotide exchange.^19^ Interaction of the Rho GTPase::RhoGDI complex with ERM proteins has also been shown to promote dissociation of the complex, as the ERM proteins can compete for binding to Rho GTPases.^20^ Phosphorylation of the Rho GTPase::RhoGDI complex allows for more precise regulation of RhoGDI release from and association with Rho GTPases. RhoGDIs can be phosphorylated to promote their dissociation from Rho GTPases, with phosphorylation of specific residues resulting in decreased affinities for specific Rho GTPases.^21,22^ Rho GTPases can also be phosphorylated, which instead results in a general increase in association with RhoGDIs.^23,24^

There are only three mammalian RhoGDI isoforms: RhoGDI1 (Arhgdiα, RhoGDIA), RhoGDI2 (Arhgdiβ, RhoGDIB, LyGDI, D4-GDI), and RhoGDI3 (Arhgdiγ, RhoGDIG). The three isoforms display different tissue expression patterns^25^ and Rho GTPase binding affinities;^26^ RhoGDI1 displays ubiquitous expression and can bind most major Rho GTPases,^27^ including RhoA, Rac1, and Cdc42. RhoGDI2 displays expression primarily in cells of hematopoietic origin^28^ and can bind many major Rho GTPases as well, but with lower affinity than RhoGDI1. RhoGDI3 is the most dissimilar, displaying expression in specific tissues such as the brain, lung, and kidneys and only associating with a small subset of Rho GTPases.^29^ While RhoGDI1 and RhoGDI2 have highly similar structures, differences in the residues in the N-terminal region and lining the hydrophobic pocket may contribute to their different Rho GTPase affinities.^30^ Aside from its specific Rho GTPase affinities, RhoGDI3 is unique in that it also has an N-terminal extension, resulting in its localization to the Golgi apparatus.^29^

Here we have examined the role of RhoGDIs in mast cell activation and degranulation. From previous studies, Rho GTPases need to be activated for mast cell degranulation.^5^ As RhoGDIs are negative regulators of Rho GTPases, we hypothesize that in mast cells, RhoGDIs inhibit proinflammatory processes such as degranulation by blocking Rho GTPase activation. However, RhoGDIs could function as positive regulators in Rho GTPase signaling via delivery to sites of active degranulation. We used RBL-2H3 cells as model mast cells and found they express both RhoGDI1 and RhoGDI2. We also hypothesize that RhoGDI1 and RhoGDI2 may have distinct roles in mast cells, based on their different binding affinities for Rho GTPases. We generated RhoGDI1 and RhoGDI2 knockdown and overexpression strains and characterized the effects on cell morphology and degranulation. These studies suggest that RhoGDI1 primarily acts as a negative regulator of Rho GTPase signaling for degranulation. While RhoGDI2 was highly expressed in RBL-2H3 cells, it had less impact on cell activation and degranulation.

## 2. Results

### 2.1 Expression pattern of mammalian RhoGDIs

Rho proteins are known to regulate mast cell proinflammatory functions including degranulation, transcriptional activation of inflammatory mediators, and proliferation.^31–33^ RhoGDIs are a class of proteins that regulate the spatial and temporal functions of many Rho proteins.^18^ The goal of this study was to define the roles of RhoGDIs in FcεRI-mediated degranulation, a proinflammatory signaling process found in mast cells. To study the function of RhoGDIs in this process, we used the rat basophilic leukemia cell line RBL-2H3 as a mast cell model.^34^ Three RhoGDI isoforms are encoded in the mammalian genome, RhoGDI1, RhoGDI2, and RhoGDI3. In mammals, RhoGDI1 is ubiquitously expressed with low tissue specificity while RhoGDI2 and RhoGDI3 have distinct expression patterns.^35^

**Fig. 1.**
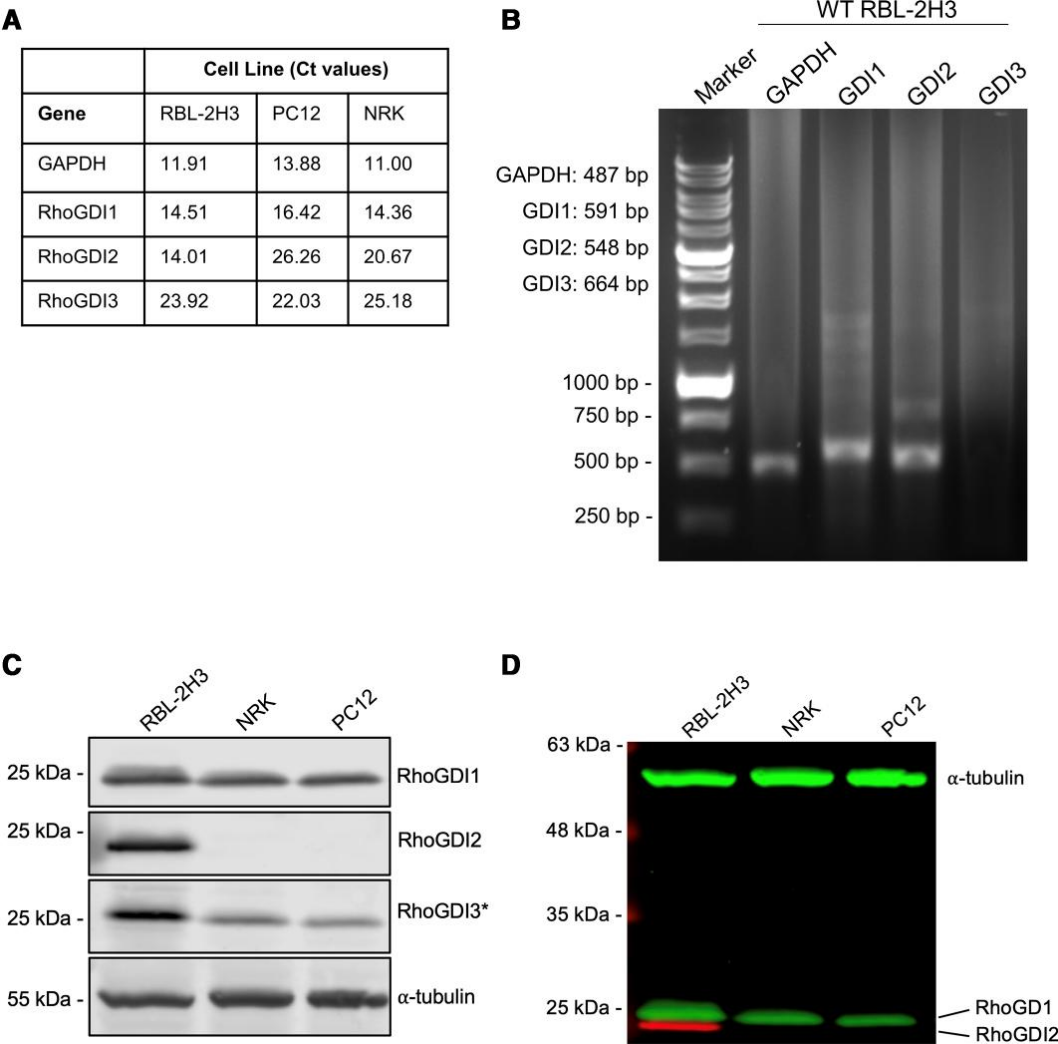
Expression of RhoGDI isoforms in various rat cell lines. A) Ct-values from qPCR analysis of RNA extracted from different rat cell lines. Data shows the relative abundance of RhoGDI mRNA with GAPDH as a reference gene. B) Agarose gel electrophoresis of RT-PCR DNA fragments showing expression of RhoGDI1 and RhoGDI2 mRNA in RBL-2H3 cells, with no detectable RhoGDI3 mRNA. C) Immunoblot for RhoGDIs in lysates from different rat cell lines. * indicates cross-reactivity of RhoGDI3 antibodies with RhoGDI1; specificity of RhoGDI antibodies is shown in Supplementary Fig. 1, Supplementary Data. D) Colored overlay of RhoGDI1, RhoGDI2, and tubulin immunoblots from *panel C*, showing the relative size of proteins with respect to each other.

To determine which RhoGDIs to study in mast cells, we examined the expression of all three RhoGDIs in RBL-2H3 cells and other mammalian cell lines of rat origin. qPCR revealed RBL-2H3 cells express high levels of RhoGDI1 and RhoGDI2 mRNA, and very low levels of RhoGDI3 (Fig. 1A). Comparison to other rat cell lines, NRK and PC12 cells only express RhoGDI1 mRNA (Fig. 1A). Analysis of RNA levels by limited RT-PCR and gel electrophoresis confirmed expression of RhoGDI1 and RhoGDI2 in RBL-2H3 cells, with no detectable levels of RhoGDI3 (Fig. 1B). Immunoblot of whole-cell lysates showed expression of RhoGDI1 protein in all cell lines tested, while RhoGDI2 was only detected RBL-2H3 cell lysates (Fig. 1C). While RhoGDI3 expression was very low by qPCR and undetectable by RT-PCR in all cell lines, immunoblot analysis with a RhoGDI3 antibody detected a peptide at the same molecular weight as RhoGDI1 (Fig. 1C). Analysis of the specificity of RhoGDI antibodies showed that RhoGDI3 antibody cross-reacts with RhoGDI1 (Supplementary Fig. 1, Supplementary Data). RhoGDI3 contains a 23 amino acid N-terminal extension that results in a molecular weight increase of ∼2 kDa. A band corresponding to the molecular weight of RhoGDI3 was not present in RBL-2H3 lysates but does appear in N2a lysates, which is a neuronal cell line (Supplementary Fig. 2, Supplementary Data). These results show that RBL-2H3 cells express RhoGDI1 and RhoGDI2, which were subjected to further investigation.

### 2.2 Intracellular localization of RhoGDIs in mast cells

**Fig. 2.**
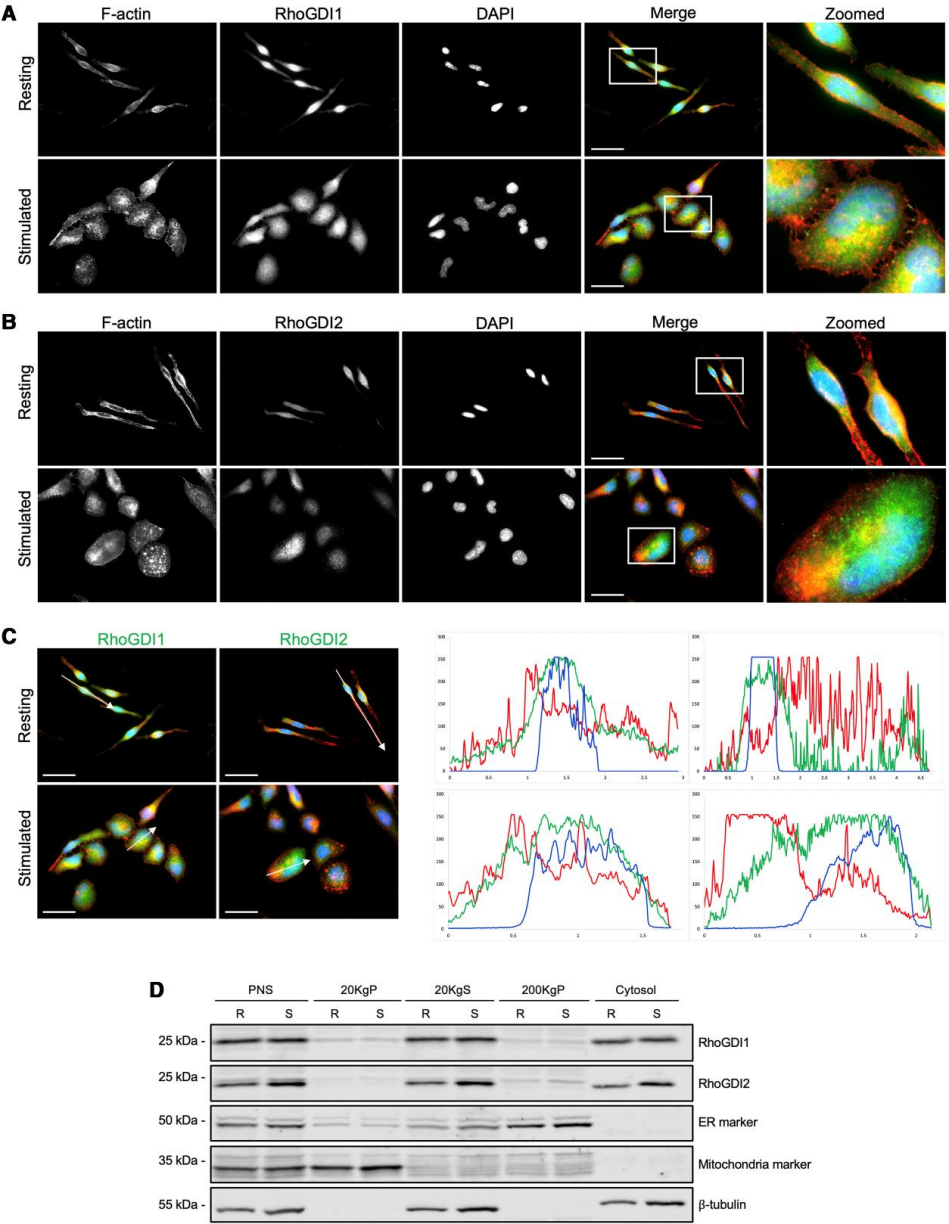
Subcellular localization of RhoGDI1 and RhoGDI2 in RBL-2H3 cells. RBL-2H3 cells were left untreated (*Resting*) or sensitized with 120 ng/mL anti-DNP-IgE for 4 h, then stimulated with 25 ng/mL DNP-BSA for 20 min at 37 °C (*Stimulated*). Cells were then fixed and stained for immunofluorescence microscopy. A, B) Resting and stimulated RBL-2H3 cells stained with antibodies against RhoGDI1 (*A*) or RhoGDI2 (*B*), F-actin (*red*, Alexa 546-phalloidin) and nuclei (*blue,* DAPI). Bar = 25 µm. RhoGDI1 and RhoGDI2 both show cytoplasmic distribution, with no significant membrane enrichment. C) The distribution of RhoGDI1 and RhoGDI2 staining in resting and stimulated RBL-2H3 cells was measured along the indicated sections (*left panels*) and shown in the profile plots (*right panels*). Bar = 25 µm. D) Differential centrifugation of RBL-2H3 cell lysates prepared from resting (*R*) and antigen-stimulated (*S*) cells. Fractions were probed for the presence of RhoGDI1 and RhoGDI2; membrane fractions were marked for ER (anti-FAM124B antibody) and mitochondria (anti-MCU antibody) and cytosol with β-tubulin. PNS, post-nuclear supernatant; 20KgP and 20KgS, pellet and supernatant fractions after 20,000 × *g* centrifugation; 200KgP and cytosol, pellet and supernatant fractions after 200,000 × *g* centrifugation.

Rho proteins are activated during antigen stimulation of mast cells.^5,31^ This could result from the delivery of Rho proteins from a soluble pool bound to RhoGDI in the cytosol and subsequently released upon antigen stimulation. RhoGDI1 and RhoGDI2 have been shown to localize to the cytosol as complexes with Rho GTPases, but upstream signals cause these complexes to translocate to membranes, releasing Rho proteins for subsequent activation.^4,18^ Therefore, we next examined the intracellular localization of RhoGDIs in mast cells. Indirect immunofluorescence microscopy of endogenous RhoGDI1 and RhoGDI2 in RBL-2H3 cells showed both RhoGDIs were predominantly cytosolic (Fig. 2A and B). Stimulation did not significantly change the localization of either RhoGDI1 or RhoGDI2. As shown in the profile plots (Fig. 2C, *green line*), RhoGDI1 and RhoGDI2 signals spread similar to cell spreading which occurs during stimulation.

We also performed subcellular fractionation using differential centrifugation to examine RhoGDI localization. RBL-2H3 lysates were separated into membrane and cytosolic fractions and analyzed by immunoblot. We found RhoGDI1 predominantly in the cytosolic fractions, while a small fraction of RhoGDI1 was also present in heavy and light membrane fractions (Fig. 2D). RhoGDI2 was found solely in the cytosolic fraction (Fig. 2D). Stimulation did not induce any observable changes in localization of either RhoGDI. These results confirm the predominantly cytosolic localization of RhoGDIs.

### 2.3 RhoGDI interactions with Rho GTPases

**Fig. 3.**
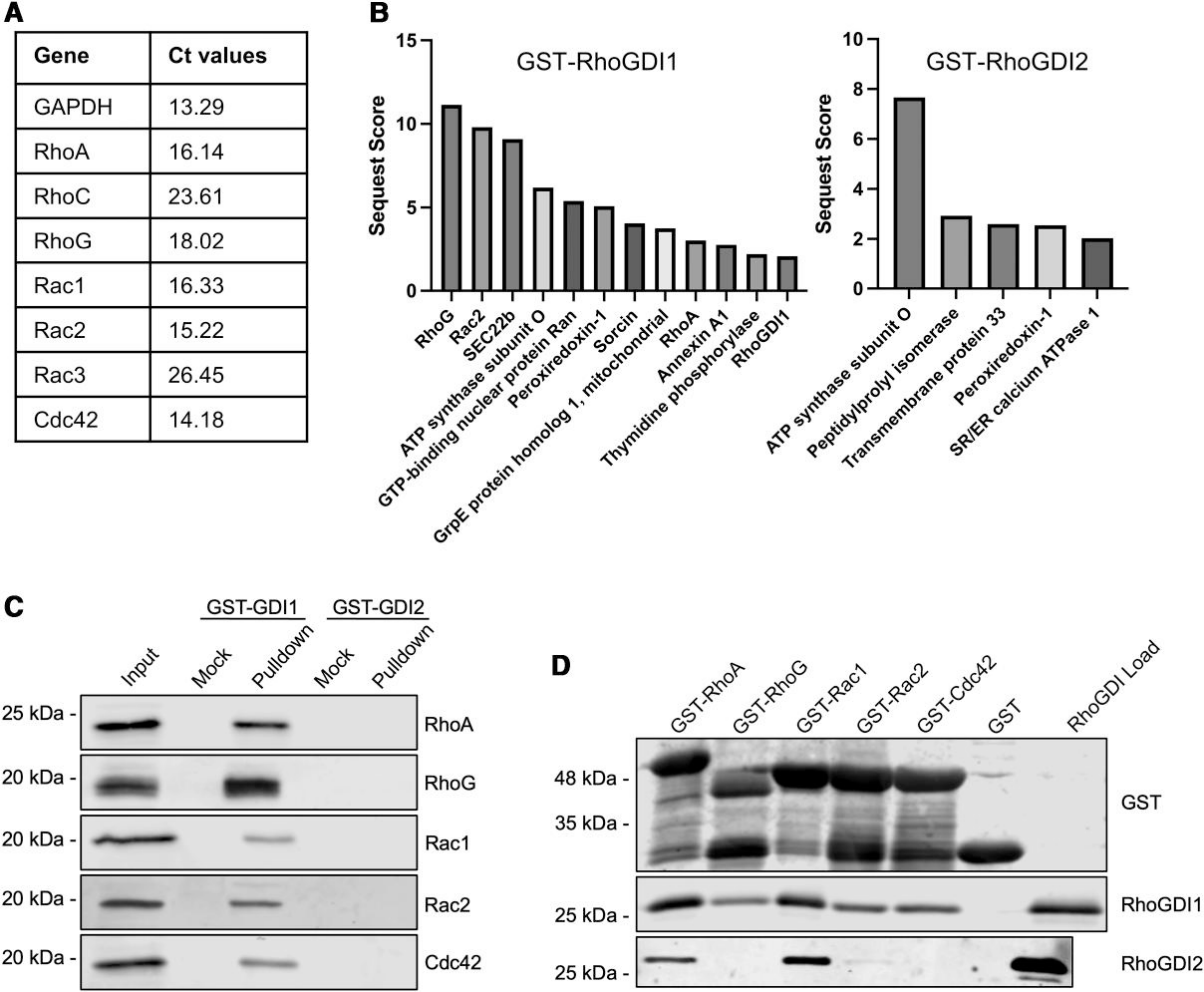
RhoGDI interactions with Rho GTPases. A) Ct-values from qPCR analysis of RNA extracted from RBL-2H3 cells. Data shows the relative abundance of various Rho GTPase mRNA with GAPDH as a reference gene. B) Analysis of endogenous Rho GTPase::RhoGDI interactions by affinity pulldown assays using GST-tagged RhoGDIs incubated with RBL-2H3 lysates. RBL-2H3 lysates were incubated with GST-RhoGDIs immobilized on glutathione beads. Affinity pulldown samples were separated by SDS-PAGE and analyzed by mass spectrometry following in-gel digestion of bands excised from the 20 to 25 kDa region. Top identified proteins were sorted in order of descending SEQUEST scores. C) Affinity pulldown samples from *B* were separated by SDS-PAGE and analyzed by immunoblotting. Lanes labeled *Mock* indicated beads incubated with lysis buffer instead of lysate. Immunoblots were probed with antibodies against RhoA, RhoG, Rac1, Rac2, and Cdc42. A control GST-only pulldown shows no background binding of Rho proteins to GST or the glutathione resin (Supplementary Fig. 3, Supplementary Data). (D) Purified His_6_-tagged RhoGDIs were incubated with GST-tagged Rho GTPases immobilized on glutathione beads. Immunoblots show interactions between Rho GTPases and RhoGDI1 and RhoGDI2. Anti-GST and anti-RhoGDI antibodies were used to detect GST-tagged Rho GTPases and His_6_-tagged RhoGDIs, respectively. RhoGDI1 interacted with all Rho GTPases with high affinity for Rac1 and RhoA while RhoGDI2 showed preferential binding for RhoA and Rac1.

We next wanted to determine the pool of Rho proteins complexed with RhoGDI1 and RhoGDI2 in mast cells. qPCR analysis showed significant mRNA levels for Cdc42, Rac2, Rac1, and RhoA, and slightly lower levels for RhoG (Fig. 3A). RhoGDI1 and RhoGDI2 immunoprecipitation showed co-immunoprecipitation of Rho proteins with RhoGDI1 only (unpublished observation).

However, antibodies against RhoGDI2 did not result in appreciable immunoprecipitation of RhoGDI2 protein. Therefore, we examined interactions of endogenous Rho GTPases by affinity pulldown assays using purified GST-RhoGDIs incubated with RBL-2H3 lysates. Analysis of the GST-RhoGDI pulldown fractions by mass spectrometry showed RhoGDI1 interacted with numerous Rho GTPases with RhoG and Rac2 showing the highest Sequest scores, while RhoA and much less Rac1 was detected (Sequest score of < 1 for Rac1) (Fig. 3B, *left panel*). RhoGDI2 pulldowns showed only background interactions (Fig. 3B, *right panel*). Immunoblot analysis revealed GST-RhoGDI1 was able to pulldown RhoA, RhoG, Rac1, Rac2, and Cdc42 from RBL-2H3 lysates (Fig. 3C). GST-RhoGDI2 was not able to pulldown any Rho GTPases even though it was previously shown to interaction with Rac1.^26^

Previous interaction studies defined the set of RhoGDI1 interactions which occurred with most Rho proteins and RhoGDI2 with a limited subset of Rac1, Rac3, and RhoC.^26^ We performed direct binding assays with purified proteins; GST-tagged Rho proteins were immobilized on glutathione resin and incubate with His_6_-tagged RhoGDIs. The Rho GTPases examined in this experiment were limited to those that are abundant in mast cells (*see* Fig. 3A). RhoGDI1 interacted with all Rho proteins tested (Fig. 3D, *middle panel*) which is consistent with results using mast cell lysates. However, the highest levels of RhoGDI1 interaction were with Rac1 and RhoA via direct binding assay while RhoG and Rac2 showed the highest binding when the analysis examined endogenous Rho GTPases. RhoGDI2 also interacted with Rac1 and to a lesser extent with Rac2 and RhoA in direct binding assays, while no interaction was observed with RhoG or Cdc42 (Fig. 3D, *bottom panel*). Differences in the levels of RhoGDI1 interaction between the two assays, and the absence of detectable Rho GTPase::RhoGDI2 interactions in mast cells, may be due to protein modifications such as isoprenylation of Rho GTPases and phosphorylation of RhoGDIs, which are known to influence their association.^21–26^ These modifications would not be present in direct binding assays which used bacterially expressed proteins.

### 2.4 Knockdown of RhoGDIs alters Rho GTPase stability

**Fig. 4.**
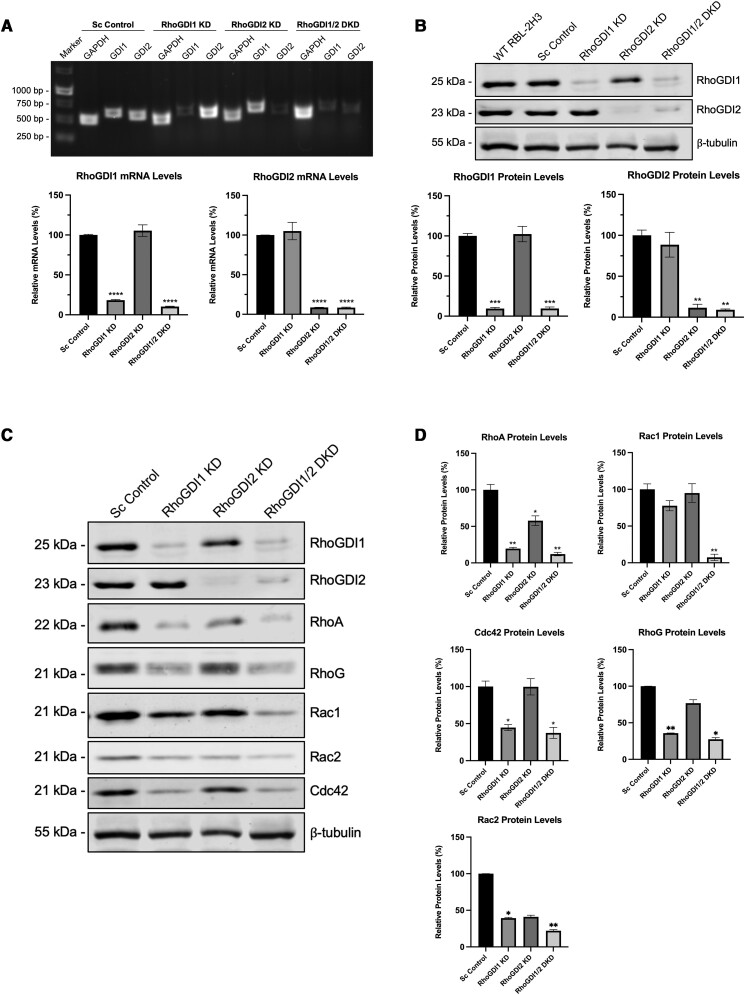
Lentiviral-mediated shRNA knockdown of RhoGDIs affects Rho GTPase stability. RNA and protein samples were prepared from RBL-2H3 RhoGDI knockdown, DKD, and scrambled control cell lines as indicated. A) Agarose gel electrophoresis of RT-PCR DNA fragments (*upper panel*) and qPCR analysis, based on 2^−ΔΔCt^ values compared to scrambled control, (*lower panel*) show a reduction of RhoGDI expression in their respective KD strains relative to control cell lines. No significant changes occurred in nontargeted RhoGDI mRNA levels. B) Immunoblots probed for RhoGDI (*upper panel*) and their quantification (*lower panel*) show a significant reduction in RhoGDI protein levels in each knockdown cell line. C, D) Immunoblots of RhoGDI KD cell lysates probed for RhoA, RhoG, Rac1, Rac2, and Cdc42 (*panel C*) and their quantification (*panel D*) show a significant reduction in Rho protein levels. Levels of reduction correlate inversely with their binding affinities for RhoGDIs; RhoA is affected by individual KD of both RhoGDIs, while Rac1 is only affected by simultaneous KD of both RhoGDIs. Cdc42 levels are only affected by RhoGDI1 KD, and RhoGDI2 KD has no effect. *P < 0.1; **P < 0.01; ***P < 0.001; ****P < 0.0001; *n* = 3.

Although RhoGDI proteins are generally considered to be negative regulators of Rho GTPases, positive regulatory roles have also been shown since they are involved in the delivery of Rho GTPase to active sites.^29,35^ To assess the role of RhoGDIs in mast cell degranulation, we generated stable RBL-2H3 cell lines with depleted expression of RhoGDI1, RhoGDI2 or both RhoGDI1 and RhoGDI2, using lentiviral transduction of specific shRNAs. Stable clones were obtained by puromycin selection and were confirmed to be deficient in RhoGDIs by gel electrophoresis of RT-PCR products and by qPCR analysis (Fig. 4A). Immunoblot analysis of whole-cell lysates revealed significantly reduced RhoGDI protein levels in respective knockdown cell lines (Fig. 4B). There was no compensatory effect observed due to single RhoGDI knockdown; e.g. knockdown of RhoGDI1 expression did not result in an increase in RhoGDI2 expression.

RhoGDIs stabilize prenylated Rho GTPases in the cytosol; therefore, we next examined if total Rho GTPase levels were affected by RhoGDI knockdown. We found RhoGDI1 and RhoGDI2 were differentially required for Rho protein stability, but depletion of both resulted in significant reduction of all Rho proteins (Fig. 4C and D). The stability of RhoA was dependent on both RhoGDI1 and RhoGDI2; RhoA protein levels were reduced following knockdown of either RhoGDI1 or RhoGDI2, although RhoGDI1 knockdown cell line had significantly lower RhoA levels. Single knockdown of either RhoGDI1 or RhoGDI2 did not significantly affect the stability of Rac1 and Rac2, but the RhoGDI1/2 double knockdown (DKD) cell line had significantly reduced levels of both. Cdc42 and RhoG stability was primarily dependent on RhoGDI1, but not RhoGDI2. This demonstrates differential roles for RhoGDIs in stabilizing Rho proteins in mast cells.

### 2.5 Knockdown of RhoGDIs affects Rho GTPase localization and activity

**Fig. 5.**
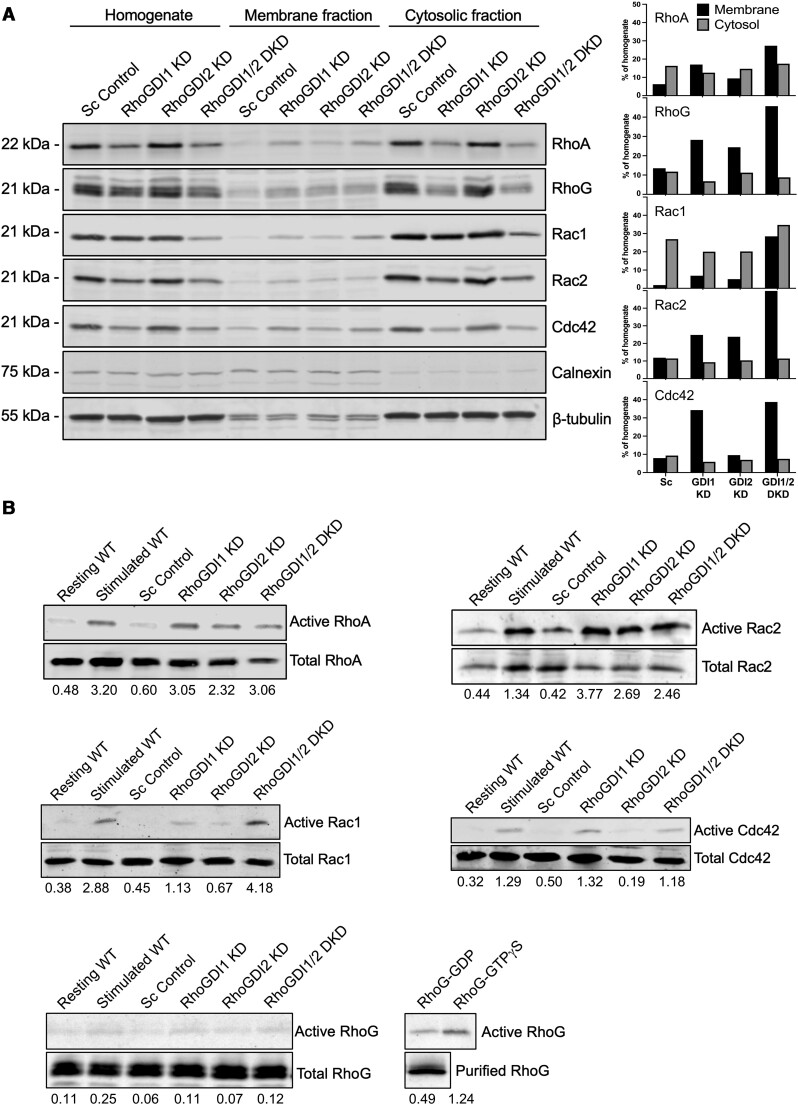
Rho GTPase subcellular localization and activity are affected by RhoGDI KD. A) Analysis of the subcellular localization of Rho proteins. Differential centrifugation was used to separate cell lysates into post-nuclear homogenate, membrane and cytosolic fractions. Calnexin (ER protein) was used as a membrane marker while tubulin was used as a cytosolic marker. Overall levels of Rho GTPases were found to be decreased in RhoGDI KD lines, and analysis of the cytosolic fraction revealed Rho GTPases were deficient in the cytosol of the RhoGDI KDs relative to the controls. RhoGDI KD lysates showed increased Rho GTPase levels associated with membranes compared to control lines (*middle four rows*). *Right panels*, levels of Rho GTPases detected in cytosol and membrane fractions were quantified by band densitometry of blots. B) Detection of active Rho proteins. Rhotekin pulldowns from RBL-2H3 lysates revealed that unstimulated RhoGDI KD cells had elevated active RhoA levels, similar to stimulated WT cells (*top left panel*). PAK1 pulldowns showed RhoGDI knockdown increased active Rac1 and Rac2 levels (*middle panels*). PAK1 pulldown showed RhoGDI1 KD and RhoGDI1/2 DKD, and not RhoGDI2 KD, increased active Cdc42 levels (*top right panel*). The ELMO pulldown did not show the presence of active RhoG in stimulated WT or RhoGDI KD cells (*bottom panel*), while it was able to detect purified RhoG bound to GTPγS. Numbers below blots indicate the percent active Rho GTPase of the total quantified by band densitometry of blots.

Isoprenylated Rho GTPases are typically associated with membranes in the absence of RhoGDI binding. Since depletion of RhoGDIs resulted in lowered levels of Rho GTPases, we next examined if the localization of the remaining Rho GTPases was affected. We separated RBL-2H3 lysates into membrane and cytosolic fractions by centrifugation of post-nuclear supernatants at 200,000 × *g*. Lower levels of Rho GTPases were detected in the cytosol when RhoGDIs were depleted, particularly in the RhoGDI1 KD and RhoGDI1/2 DKD cell lines (Fig. 5A, *Cytosolic fraction*), indicating that the overall decrease in Rho GTPase levels seen in the homogenate is due to depletion of Rho GTPases from the cytosol. Likewise, Rho GTPases were associated with membranes to a greater degree in the RhoGDI1 KD and RhoGDI1/2 DKD cell lines compared to the scrambled control line (Fig. 5A, *Membrane fraction*). Association with membranes may protect Rho GTPases from degradation in the absence of RhoGDIs, indicated by the higher concentration of Rho GTPases in membrane fractions. Blots were quantified for Rho protein levels in cytosol and membrane fractions (Fig. 5A, *left panels*). The control strain showed higher levels in the cytosol for RhoA and Rac1 or equal distribution for RhoG, Rac2, and Cdc42 (Fig. 5A, *Sc left panels*). RhoGDI depletion, particularly in the RhoGDI1/2 DKD strain, resulted in Rho GTPase enrichment in the membrane fraction (Fig. 5A, *GDI1/2 DKD left panels*).

Rho GTPases are activated at membranes following their release from RhoGDIs, so we determined whether the shift in observed Rho GTPase localization altered their activity. In the active GTP-bound state, Rho GTPases interact with specific downstream effectors. We affinity isolated active Rho proteins using probes created from the Rho-binding domains of these downstream effectors and analyzed the pulldowns by immunoblot.^36^ Unstimulated WT and scrambled control cells displayed low levels of GTP-bound Rho GTPases, and stimulation of WT cells increased the GTP-bound levels of all Rho GTPases except RhoG (Fig. 5B). In the RhoGDI KD cell lines, we detected high levels of active Rho GTPases even when unstimulated. While active Rac1 was not elevated in RhoGDI1 KD or RhoGDI2 KD, active Rac1 was significantly increased in the RhoGDI1/2 DKD (Fig. 5B, *middle panel*). Active RhoA and Rac2 were elevated in all unstimulated RhoGDI KD cell lines compared to the scrambled control (Fig. 5B, *top panels*). Similar to RhoA, active Cdc42 was increased in RhoGDI1 KD and RhoGDI1/2 DKD. However, RhoGDI2 KD did not affect active Cdc42 levels, likely due to their lack of interaction. Active RhoG was not detected in any samples; the RhoG activation assay probe (GST-ELMO) was able to detect purified RhoG bound to GTPγS, which indicated that the assay was working (Fig. 5B, *bottom panel*).

### 2.6 Knockdown of RhoGDIs affects mast cell morphology and degranulation

**Fig. 6.**
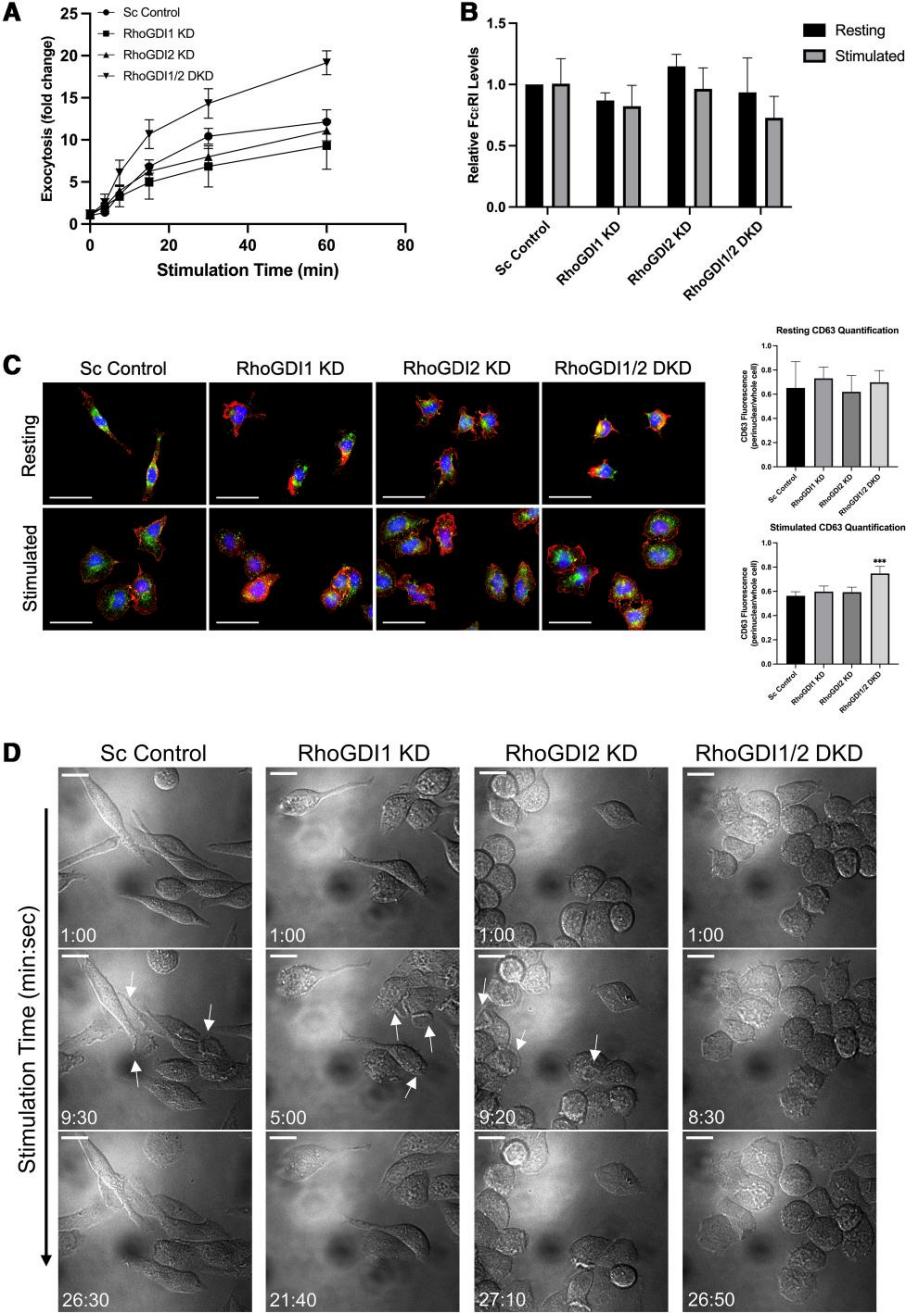
RhoGDI KD increases mast cell degranulation and activated cell morphology. A) Degranulation of RhoGDI KD cell lines was measured by β-hexosaminidase released into supernatants. IgE-sensitized cells were stimulated with DNP-BSA and extracellular β-hexosaminidase was compared to the total of intracellular and extracellular β-hexosaminidase. All values were normalized to the resting state scrambled control. Fold change was calculated as the ratio of stimulated/resting. Knockdown of both RhoGDIs significantly increased degranulation (*P* = 0.016; *P* = 0.0110; *P* = 0.0006 at 15, 30, and 60 min respectively, comparing RhoGDI1/2 DKD to Sc control; *n* = 5). B) Analysis of FcεRI surface levels in RhoGDI KD strains. Cells were left resting or stimulated for 30 min, then fixed and stained with an FcεRIα antibody and analyzed by flow cytometry. All values were normalized to the resting state scrambled control. Stimulation slightly decreased surface FcεRI levels for all strains. However, no significant differences were observed in FcεRI surface levels between strains. Flow cytometry was done on four biological replicates for each strain (sample data are provided in Supplementary Fig. 4, Supplementary Data). C) Cells were imaged by widefield microscopy and stained for nuclei (*blue*), F-actin (*red*), and CD63+ granules (*green*) before (*Resting*) and after stimulation. Bar = 25 µm. In the resting state, Sc control cells are elongated, however RhoGDI KD cells are condensed. When stimulated, all cells from all strains transition to an activated state of spread-out and flattened cells. CD63+ granules were localized to the perinuclear region in all strains in their resting states. Upon stimulation, CD63+ granule staining appeared more punctate throughout the cell body, most clearly seen in the RhoGDI2 KD and RhoGDI1/2 DKD strains. *Right panels* show quantification of CD63 fluorescence signal in the perinuclear region versus the whole cell. RhoGDI1/2 DKD had increased CD63 signal in the perinuclear region relative to the whole cell compared to the other strains. ***P < 0.001; *n* ≥ 6 cells. D) Still frames from live cell imaging videos of stimulated RBL-2H3 cells. Cells were imaged by differential interference microscopy. Bar = 20 µm. Sc cells formed large dorsal ruffles; however, RhoGDI1 KD and RhoGDI2 KD formed only small dorsal ruffles (*arrows*). RhoGDI1/2 DKD did not form dorsal ruffles. All cells returned to the resting state after prolonged imaging except RhoGDI1/2 DKD cells which maintained a stimulated state. Videos are available at the following DOIs (shown at 60× speed): Video 1: 10.6084/m9.figshare.25555983. Video 2: 10.6084/m9.figshare.25492906. Video 3: 10.6084/m9.figshare.25492915. Video 4: 10.6084/m9.figshare.25492927.

Rho GTPase activity was previously found to enhance antigen-stimulated degranulation in mast cells.^31,37,38^ Knockdown of RhoGDIs affected the activation states of Rho GTPases; therefore, we next examined how these changes in Rho activity affected degranulation and activation of mast cells. We found that RBL-2H3 strains depleted of either RhoGDI1 or RhoGDI2 did not show a significant effect on degranulation when compared to the control strain (Fig. 6A). However, the RhoGDI1/2 DKD showed significant increases in degranulation levels compared to the scrambled control strain, with fold changes of 10.7, 14.3, and 19.2 at 15, 30, and 60 min respectively, (*P* = 0.016; *P* = 0.0110; *P* = 0.0006) (Fig. 6A). RhoGDI KD cell lines showed similar levels of FcεRI receptor on the surface of cells (Fig. 6B), indicating that differences in degranulation were likely not due to receptor signaling. This suggests that RhoGDIs act as a negative regulator of Rho protein function for degranulation downstream of membrane proximal signaling.

To determine if changes in degranulation levels were due to alterations in F-actin remodeling, cell morphology and granule distribution were examined by immunofluorescence microscopy. In the resting state, scrambled control cells are elongated and tube-like, and when antigen-stimulated undergo cytoskeletal rearrangement causing cell spreading and a flattened activated state (Fig. 6C, *Sc Control*). Depletion of RhoGDIs resulted in the loss of the elongated morphology of normal resting cells, with RhoGDI-depleted cells instead adopting an irregular and compact morphology while cell spreading after stimulation appeared normal compared to the scrambled control (Fig. 6C, *RhoGDI1 KD* and *RhoGDI2 KD*). The resting morphology of the RhoGDI1/2 DKD appeared similar to the individual RhoGDI KDs, but after stimulation the DKD cells showed a more flattened morphology (Fig. 6C, *RhoGDI1/2 DKD*). Granule distribution was visualized by anti-CD63 staining. CD63+ granules mainly localized to the perinuclear region in resting cells of all strains, although some granules in the control cells were distributed further away from the cell body (Fig. 6C, *Resting*). Following stimulation, CD63+ granule staining appeared more punctate and was distributed throughout the cell body, seen most clearly in the RhoGDI1/2 DKD strain (Fig. 6C, *Stimulated*). Quantification of CD63+ granule distribution by ImageJ analysis showed no differences in distribution prior to stimulation, while an increase in perinuclear distribution was observed in the RhoGDI1/2 DKD strain after stimulation (Fig. 6C, *right panels*). This may be due to an overall increase in the number of granules that have undergone exocytosis in this strain, resulting in a decrease in mobile granules left in the cytoplasm.

The RhoGDI KD strains were also examined by live cell imaging for differences in morphological changes in response to stimulation. The control strain formed large circular dorsal ruffles 9 to 10 min after antigen stimulation (Fig. 6D, *Sc Control*, Video 1). The RhoGDI1 KD strain formed dorsal ruffles earlier at 5 to 8 min after stimulation, although these were much smaller in size compared to the control stain (Fig. 6D, *middle panels*; Videos 2 and 3, respectively). In contrast, the RhoGDI1/2 DKD did not form any dorsal ruffles upon stimulation but still adopted the stimulated phenotype (Fig. 6D, Video 4). After extended imaging, the control and individual RhoGDI KD strains returned to a resting state (Videos 1–3) while the RhoGDI1/2 DKD maintained a stimulated state (Video 4). This suggests that the increased levels of degranulation from the RhoGDI1/2 DKD strain may be due to a delay in downregulation of antigen stimulation.

### 2.7 RhoGDI KD drug sensitivity

**Fig. 7.**
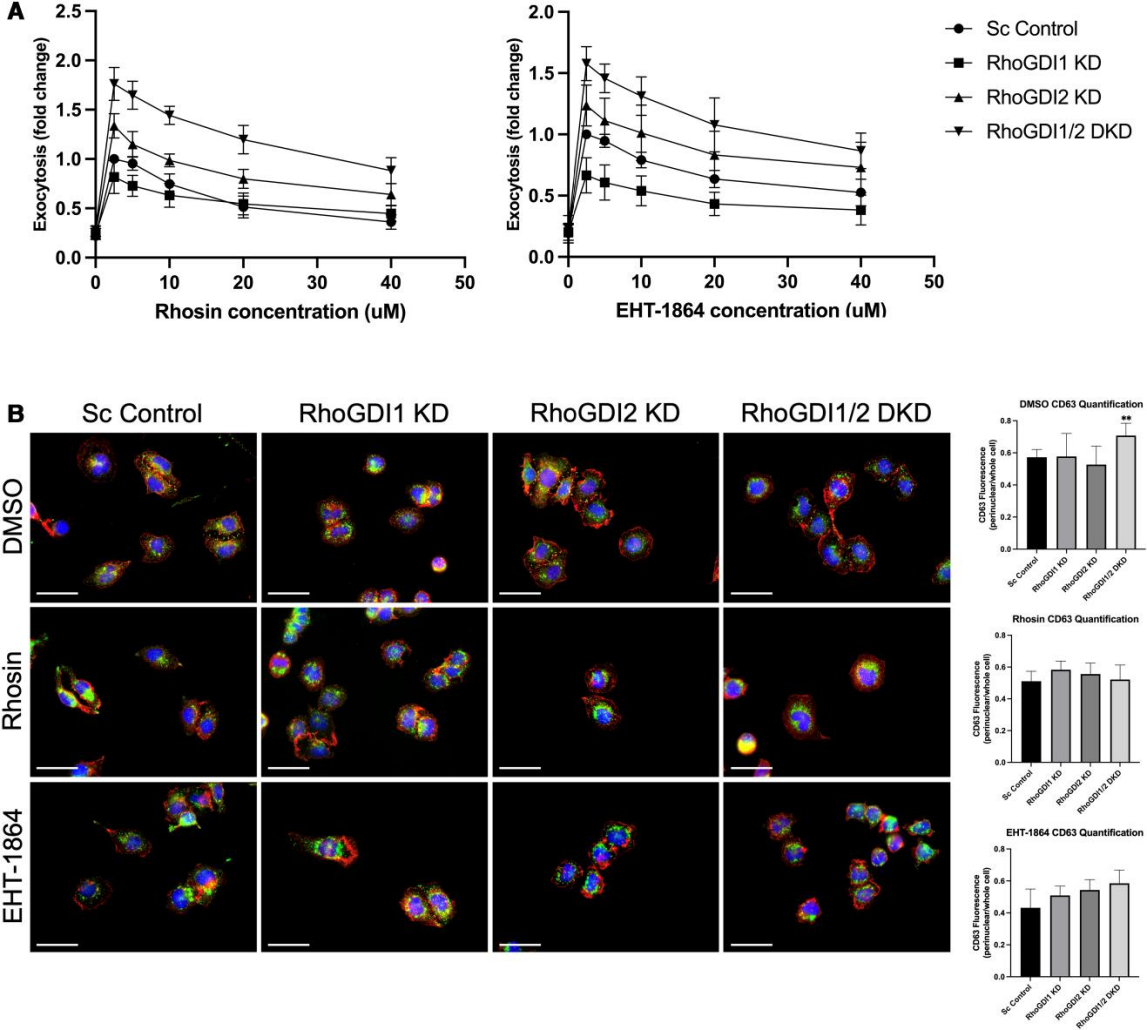
Knockdown of RhoGDIs does not affect sensitivity of cells to Rho inhibitors. RhoGDI KD strains were incubated with Rhosin or EHT-1864 for 15 min at the indicated concentrations prior to stimulation. Degranulation assays were performed following 30 min of stimulation and exocytosis levels were normalized to the scrambled control treated with 2.5 μM of Rho drug. Fold change was calculated as the ratio of drug treated, 30 min stimulation/resting. A) Rhosin inhibited degranulation of all strains in a concentration-dependent manner, although no significant differences were observed within each strain with regards to the dose response of Rhosin at increasing concentrations. EHT-1864 inhibited degranulation in all strains in a concentration-dependent manner with no significant differences in dose response (*n* = 5). B) Cells were pretreated with Rhosin or EHT-1864 for 15 min prior to antigen stimulation for 20 min and imaged by widefield microscopy. Cells were stained for nuclei (*blue*), F-actin (*red*), and CD63+ granules (*green*). Bar = 25 µm. Cell morphology was not noticeably affected after RhoA or Rac1 inhibition. Quantification of CD63 fluorescence signal in the perinuclear region versus the whole cell showed no significant differences between strains across all treatments. **P < 0.01; *n* ≥ 6 cells.

Knockdown of RhoGDIs was found to affect levels of Rho GTPases and enhance degranulation; therefore, we next examined whether sensitivity of degranulation to Rho inhibitors was also affected in RhoGDI KD strains. The Rho drugs Rhosin and EHT-1864 were used to inhibit RhoA and Rac1, respectively. Incubation of cells with Rhosin prior to stimulation inhibited degranulation in a concentration-dependent manner for all strains, with significant inhibition of degranulation at the highest drug concentration (Fig. 7A, *left panel*). None of the RhoGDI KD strains showed any noticeable differences in sensitivity to Rhosin compared to the control strain. Incubation of EHT-1864 also reduced degranulation levels in a concentration-dependent manner; sensitivity to higher concentrations of EHT-1864 was not significantly affected in the RhoGDI KD strains compared to the control (Fig. 7A, *right panel*). These results indicated that although Rho GTPase levels are reduced in RhoGDI KD strains, these cells still utilize the same Rho-signaling pathway in degranulation as the control cells.

Control and RhoGDI KD strains were also imaged after stimulation in the presence of RhoA and Rac1 inhibitors. F-actin staining revealed a change in sensitivity to the RhoA inhibitor, Rhosin. While cell spreading was reduced in the scrambled control strain after Rhosin treatment, RhoGDI KD strains maintained the ability to spread after stimulation (Fig. 7B). This suggests that Rho protein function for morphological transition was less sensitive to drug. This could be due to the increase in membrane-localized or -activated Rho proteins that occur in RhoGDI KD strains (*see* Fig. 5). Quantification of granule distribution showed a small increase in perinuclear granules only in the RhoGDI1/2 DKD strain compared to the control (Fig. 7B, *left panels*). This is likely due to a decrease in overall granule numbers in the whole cell since degranulation was higher in the RhoGDI1/2 DKD strain. Taken together these results show that while overall Rho proteins were reduced when RhoGDIs were depleted, the remaining Rho proteins can still provide for efficient signaling.

### 2.8 Effect of overexpression of RhoGDIs on Rho GTPase stability

**Fig. 8.**
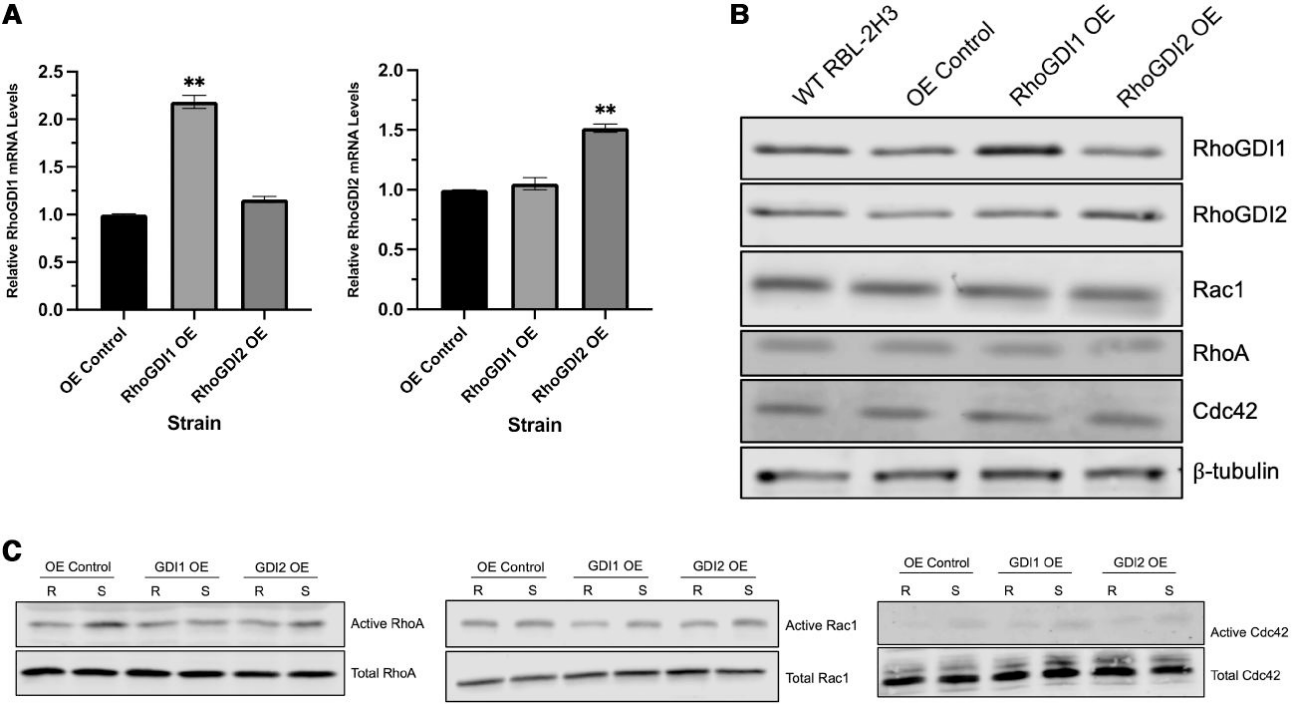
Overexpression of RhoGDIs does not affect Rho GTPase levels or activity following stimulation. A) Quantitative PCR showing significant overexpression of RhoGDIs in their respective strains without affecting the expression of the other RhoGDI. Data shown are 2^−ΔΔCt^ values based on comparison to empty vector control (OE control). **P < 0.01; *n* = 4. B) Immunoblots of RhoGDI overexpression lysates showing increases in RhoGDI1 and RhoGDI2 protein without affecting Rho GTPase levels. C) Analysis of active Rho GTPases in control and RhoGDI overexpression cell lines. Overexpression of RhoGDIs did not noticeably affect activation of RhoA (*left panel*), Rac1 (*middle panel*), or Cdc42 (*right panel*), with active levels increasing following antigen stimulation in all strains.

Since knockdown of RhoGDIs affected Rho GTPase stability and enhanced mast cell degranulation, we sought to determine the effects of RhoGDI overexpression in mast cells. RBL-2H3 cell lines overexpressing either RhoGDI1 or RhoGDI2 were generated by lentiviral transduction of overexpression vectors followed by puromycin selection to obtain stable clones. Quantitative PCR confirmed RhoGDI mRNA levels were significantly elevated in their respective cell lines (Fig. 8A). Immunoblot analysis of RhoGDI overexpression strains showed increased RhoGDI protein levels while levels of Rho GTPases were relatively similar across all strains (Fig. 8B). We next examined if overexpression of RhoGDIs could inhibit Rho GTPase activity in stimulated RBL-2H3 cells by conducting Rho activation assays. We found that overexpression of RhoGDIs did not noticeably affect the levels of active Rac1, RhoA, or Cdc42 following stimulation when compared to control cells (Fig. 8C). Based on these results, RhoGDI overexpression does not affect Rho GTPase protein levels and does not inhibit the activation of Rho GTPases in the presence of stimulatory signals.

### 2.9 Effect of RhoGDI overexpression on degranulation and cell morphology

**Fig. 9.**
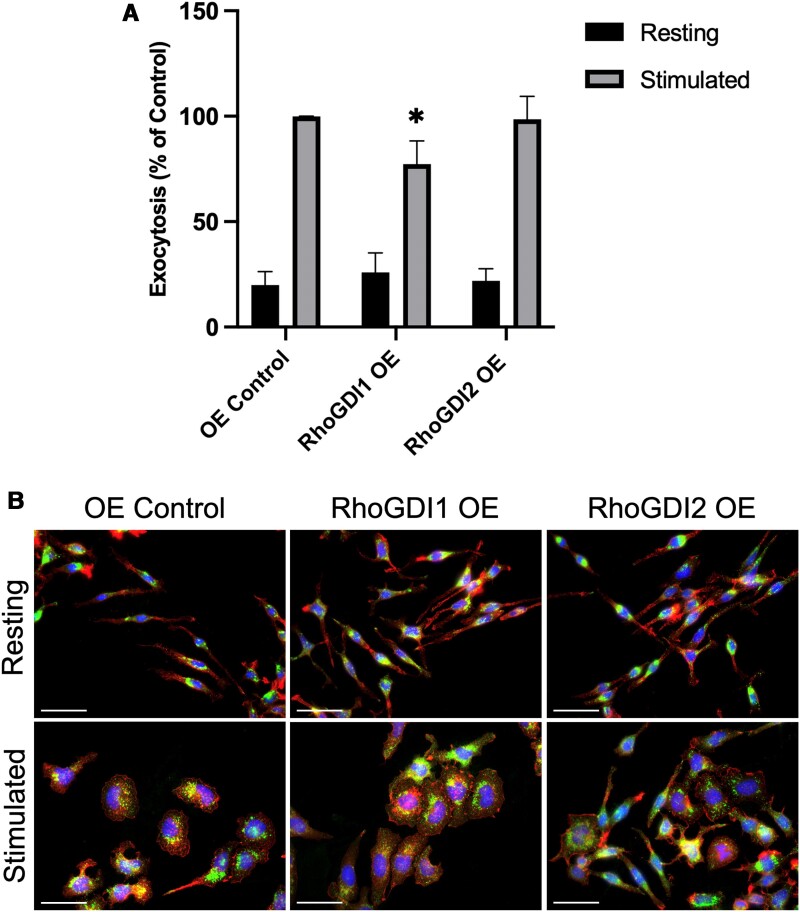
RhoGDI1 overexpression inhibits mast cell degranulation but does not affect cell morphology. A) Exocytosis levels of RhoGDI overexpression strains were analyzed by degranulation assays following 30 min of antigen stimulation. The stimulated exocytosis levels of the OE control was set as 100% and all resting and stimulated exocytosis levels were expressed as a percentage of this value. Overexpression of RhoGDI1 specifically inhibited degranulation while overexpression of RhoGDI2 did not result in a significant difference (*P < 0.05; *n* = 5). B) Control and RhoGDI overexpression cells were left resting or antigen-stimulated for 30 min then imaged for nuclei (*blue*), F-actin (*red*), and CD63+ granules (*green*). Bar = 25 µm. Overexpression of either RhoGDI1 or RhoGDI2 did not noticeably alter cell phenotypes compared to the control. CD63+ granule distribution appeared similar between all strains, with granules localized to the perinuclear region in the resting state and spreading throughout the cell body following stimulation.

Unlike knockdown of RhoGDIs, overexpression of RhoGDIs did not affect Rho GTPase levels or their ability to activate following antigen stimulation. To confirm RhoGDI overexpression did not alter other mast cell functions, we examined the degranulation ability and morphology of RhoGDI overexpression strains. RhoGDI overexpression cells were examined by degranulation assays following a 30-min stimulation period. We found that RhoGDI1 overexpression decreased degranulation by 23% ±4.2% compared to the control, while RhoGDI2 overexpression had no effect on degranulation (Fig. 9A). When we examined the cell morphology of the RhoGDI overexpression strains, we did not observe any noticeable differences between the control strain and the overexpression strains. Both RhoGDI overexpression strains in the resting state displayed the same elongated morphology of normal RBL-2H3 cells (Fig. 9B). Following stimulation, all cells were able to spread and flatten, with no discernible differences between the stimulated phenotypes of the different strains. Granule distribution was also visualized using CD63 staining, and no differences were seen between the control and RhoGDI overexpression strains. CD63+ granules were concentrated around the perinuclear region in the resting state of all cells, and spread out to adopt a more punctate pattern in stimulated cells (Fig. 9B). This was unexpected for the RhoGDI1 overexpression strain since degranulation was reduced as determined by biochemical assay; therefore, to correlate with this result it would be expected that granule should have remained more perinuclear. Our observations indicate that overexpressing RhoGDI1 had a minor effect on mast cell degranulation. This was expected since RhoGDI1 binds and sequesters most Rho proteins. More profound inhibitory effects were not observed when RhoGDIs were overexpressed which is likely due to the abundance of endogenous RhoGDIs. Stimulatory signals would likely induce Rho signaling even when Rho proteins are bound to RhoGDI1 since the normal mechanism results in their release and membrane delivery.^25^

## 3. Discussion

Mast cell degranulation is a critical step in the early phase of hyper-responsiveness in allergic diseases and Rho GTPase signaling has been established as a necessary step in degranulation.^39^ The Rho GTPases RhoA, Rac1, and Cdc42 have all been shown to have roles in mast cell degranulation through pharmacologic inhibition or genetic manipulation.^5,31,37,40^ Our goal was to determine how Rho GTPases are potentially regulated during FcεRI-mediated degranulation by examining their known regulators. RhoGDIs are a class of Rho regulators originally identified as inhibitors of Rho GTPases, as they bind Rho GTPases to prevent the exchange of GDP for GTP and maintain them in an inactive state.^25^ However, further studies have shown that RhoGDIs have the potential to act as both negative and positive Rho regulators depending on the specific cell type and process.^12,14,18^ Here we use genetic manipulation to show that RhoGDIs act as negative regulators of Rho signaling during mast cell degranulation.

We first confirmed RBL-2H3 cells expressed the RhoGDI1 and RhoGDI2 isoforms and not RhoGDI3 (Fig. 1). RhoGDI2 is predominantly expressed in hematopoietic cells,^28^ indicating that it may have a role in processes specific to those cell types. However, the structures of RhoGDI1 and RhoGDI2 are highly similar,^30^ suggesting they may also have redundant roles. We sought to determine the distinct roles of both RhoGDI isoforms in mast cell degranulation. Even though RhoGDI1 and RhoGDI2 both localize to the cytosol, they mainly differ in their N-terminal regions which may dictate how they interact with different Rho GTPases in mast cells.^12,41^ We found RhoGDI1 interacted with several Rho GTPases including RhoA, RhoG, Rac1, and Rac2; by mass spectrometry analysis RhoG and Rac2 were also identified as the predominant interactors within cells (Fig. 3). RhoGDI2 interactions were not detected in mast cell lysates and were limited to Rac1 in direct binding assays. This is similar to other in vitro studies that have shown RhoGDI1 to interact with Rac1, RhoA, and Cdc42 while RhoGDI2 interacts with Rac1.^26,42^ The lack of detectable interactions between mast cell endogenous Rho GTPases and RhoGDI2 may also be due to protein modifications. Rho protein isoprenylation may contribute to binding affinity between RhoGDIs and Rho GTPases which may result in the discrepancies we observed in the levels of binding between our assays as the direct binding assay used unprenylated Rho proteins.^26^ Additionally, RhoGDI phosphorylation has been shown to contribute to the regulation of Rho GTPase binding and release.^21–25^

The localization of Rho GTPases to their target membranes is essential for their proper function. Rac1 and RhoA activity has been observed at the leading edge of migrating cells, with RhoA also acting at the contractile tail.^15,43^ Since RhoGDIs are involved in shuttling Rho GTPases to membranes, their localization may also be integral to their function. We found both RhoGDI1 and RhoGDI2 were cytosolic with no noticeable change in localization following stimulation. Using differential centrifugation, we found both RhoGDIs mainly in the cytosolic fraction, although small amounts of RhoGDI1 and RhoGDI2 were present in fractions with ER proteins (Fig. 2D). The lack of detectable membrane localization may be due to transient interactions with Rho GTPases at membranes and the predominantly large cytosolic pool where they sequester inactive Rho GTPases.^18,44^

The chaperone function of RhoGDIs has been previously established; they bind the switch region through protein interactions and sequester the isoprenoid moiety of Rho GTPases via a C-terminal β-barrel which results in protection from degradation in the cytosol.^13,29,35^ We found that knockdown of RhoGDI1 and RhoGDI2 expression in mast cells also resulted in reduced Rho protein levels (Fig. 4C). The decrease in specific Rho GTPase levels in each RhoGDI knockdown strain corresponded with their Rho-binding affinities. Knocking down RhoGDI1 alone reduced RhoA, RhoG, and Cdc42 levels, indicating that RhoGDI1 interacts with these Rho GTPases in mast cells and reducing RhoGDI1 results in their degradation (Fig. 4C and D). Knocking down RhoGDI2 alone only slightly reduced RhoA levels, suggesting a weak level of interaction. The RhoGDI1/2 DKD was the only strain that had significantly reduced Rac1 and Rac2 levels. Rac1 is a binding partner for both RhoGDIs, so reduction of one RhoGDI may be compensated for by the other RhoGDI to protect Rac1 from degradation. Only when both RhoGDIs were simultaneously knocked down was Rac1 degraded. Rac2 is 93% identical to Rac1, and should typically function similarly.

Rho GTPases are essential for cell survival and proliferation, yet the RhoGDI1/2 DKD had significantly reduced levels of all Rho GTPases examined and remained viable.^45^ However, the remaining Rho GTPases showed a shift in their levels from the cytosol to membranes (Fig. 5A). In the absence of RhoGDI binding to the Rho GTPase isoprenoid moiety, it is likely that association with membranes protects Rho GTPases from degradation in the cytosol. Past studies have shown that Rho GTPase mutants deficient in RhoGDI binding accumulate mainly in ER fractions with low levels at the plasma membrane,^35^ which is similar to our findings. Rho GTPases on membranes are activated by GEFs,^46^ and we observed increased levels of GTP-bound Rho GTPases in the RhoGDI knockdown strains even in the absence of cell stimulation (Fig. 5B). Only the RhoGDI1/2 DKD showed a considerable increase in active Rac1, likely due to the similar binding affinities to both RhoGDIs. A study of cancer cells with RhoGDIs knocked down also reported increased Rho GTPase activation accompanied with their translocation from the cytosol to membrane fractions.^47^ We believe that cells lacking RhoGDIs have higher Rho GTPase activity because the remaining Rho GTPases left on membranes are increasingly subjected to activation by the RhoGEFs. Interestingly, RhoG showed significant interactions with RhoGDI1 (Fig. 3B–D). RhoG was shown to regulate granule exocytosis in platelets,^48^ and thus could also play a similar role in mast cells. However, RhoG was not activated by antigen stimulation and depletion of RhoGDIs did not increase active RhoG levels (Fig. 5B). Therefore, it seems that the regulation of granule exocytosis is cell-type specific.

We next examined how RhoGDI depletion affects mast cell activation and degranulation in response to antigen stimulation. Knockdown of both RhoGDIs (RhoGDI1/2 DKD) was required to obtain a significant increase in degranulation after antigen stimulation, while knockdown of one RhoGDI did not have a significant effect (Fig. 6A). This is likely due to the higher levels of active Rho GTPases in the absence of both RhoGDIs, along with elevated levels on membranes. In the event of stimulatory signals, active Rho GTPases at their target sites may be poised to act, resulting in a robust degranulation response i.e. prolonged due to the lack of RhoGDIs to halt Rho GTPase membrane signaling. These results also suggest that Rac1 potentially plays a more significant role in degranulation than Rac2, RhoA, or Cdc42; active Rac1 levels were significantly elevated only in the RhoGDI1/2 DKD strain while these other Rho GTPases were elevated in single KD strains (Fig. 5B). Therefore, the presence of active Rac1 correlates with enhanced degranulation.

Cell morphology can be used to indicate activation state.^5,7,8^ RBL-2H3 cells transition from a tube-like morphology when resting (unstimulated) to a flattened, spread-out morphology when stimulated (Fig. 6D, *Sc Control*, Video 1). It is possible that cells require a certain level of Rho GTPase inactivation to maintain a resting morphology.^49^ When this balance is disrupted, it could result in the morphological defects we observed in the knockdown strains. We saw cell spreading occur in all knockdown strains after antigen stimulation, indicating that the active fraction of Rho GTPases was sufficient to mediate cytoskeletal rearrangements (Fig. 6B). Similar to our own observations, a study of renal mesangial cells also found that overall Rho GTPase levels were decreased following RhoGDI knockdown, but with an increase in their active levels.^50^ However, knockdown of RhoGDI in mesangial cells reduced cell spreading following adhesion while we observed no defects in cell spreading with our RhoGDI knockdown cells. The signaling cascade induced by antigen binding to FcεRI is likely sufficient to promote spreading with the remaining Rho GTPases.

RBL-2H3 cells form circular dorsal ruffles on their surface when undergoing stimulation.^2,5^ The formation of these F-actin-rich structures are driven by active Rho GTPases and flatten out into lamellipodia and lead to cell spreading.^51^ In our control strain, we observed the formation of large circular dorsal ruffles before cells spread out and flattened (Fig. 6d). All RhoGDI KD strains were able to spread but only formed smaller ruffles in the single RhoGDI knockdowns and no ruffles in the RhoGDI1/2 DKD. This could be due to the smaller pool of available Rho GTPases to drive these remodeling events. After a prolonged period of stimulation, the RhoGDI1/2 DKD did not return to a normal resting morphology and instead remained flattened (compare Videos 1–4). This could be due to the lack of RhoGDIs to extract active Rho GTPases from membranes, allowing for continuous mediator release. Hence, a critical step in mast cell deactivation might be Rho GTPases extraction by RhoGDIs, allowing cells to return to the resting state.

Overall, RhoGDIs act as negative regulators of Rho signaling during mast cell degranulation by maintaining an inactive cytosolic pool of Rho GTPases. Loss of RhoGDIs resulted in a shift in localization of Rho GTPases from the cytosol to membranes, allowing for their prolonged activation and promoting degranulation.

## 4. Materials and methods

### 4.1 Cell culture and stimulation

RBL-2H3 cells (ATCC) were grown in MEM (Sigma) supplemented with 10% heat-inactivated fetal bovine serum (Gibco), 100 units/mL penicillin, 100 μg/mL streptomycin, and 0.25 μg/mL amphotericin B at 37 °C in a 5% CO_2_ incubator. Cells were sensitized by incubation with 120 ng/mL anti-DNP-IgE (Sigma) for 3 to 5 h in MEM. Sensitized cells were stimulated by incubation with 40 ng/mL DNP_∼30_-BSA (ThermoFisher) in HEPES-Tyrode's buffer (HTB; 25 mM HEPES pH 7.4, 120 mM NaCl, 5 mM MgCl_2_, 1.5 mM CaCl_2_, 1 g/L glucose, 1 g/L BSA) for 30 min. Unstimulated cells (resting cells) were incubated with HTB without DNP-BSA.

### 4.2 Lentivirus production and transduction

RhoGDI expression was knocked down using lentivirus-mediated shRNA interference. The shRNAs targeting RhoGDI1 and RhoGDI2 mRNA were derived from the pGFP-C-shLenti shRNA vector (OriGene) with puromycin selection cassette and GFP reporter. Lentiviral particles made with overexpression plasmids for *RhoGDI1* (pLV[Exp]-EGFP:T2A:Puro-EF1A > rArhgdia) or *RhoGDI2* (pLV[Exp]-EGFP:T2A:Puro-EF1A > rArhgdib) and the empty vector (pLV[Exp]-EGFP/Puro-EF1A > ORF_stuffer) were ordered from VectorBuilder. Lentiviruses were produced in HEK-293T cells using the second generation lentiviral system. HEK-293T cells were grown in a 10 cm plate and incubated with 1 mL of Opti-MEM containing 9 µg of transfer plasmids with the shRNAs, 6 µg of psPAX2 packaging plasmid, 3 μg of pMD2.G envelope plasmid, and 72 μL of PEI (1 μg/mL). After 18 h, the media was replaced with 10 mL of MEM. Viral supernatants were then harvested 36 h later. Viral transduction was performed by incubating 500,000 cells grown in 2 mL of MEM with viral supernatant at an MOI of 10 and 10 μg/mL of polybrene for 24 h. To create stable cell strains, 1.5 μg/mL puromycin was added for 48 h for selection. Colonies were then expanded in MEM containing 0.5 μg/mL puromycin and knockdown or overexpression efficiency was assessed by qPCR.

**Table:** 

shRNA oligos		
Targeted gene	shRNA oligo sequence	Targeted region
Scrambled	GCACTACCAGAGCTAACTCAGATAGTACT	N/A
ARHGDIA	CTGTCCTGGGAGTGGAATCTCACCATCAA	644 to 672
ARHGDIB	ACCACAACAAGTCCTTCTTCACCGATGAT	585 to 613

### 4.3 Analysis of mRNA levels by RT-PCR and qPCR

Expression levels of RhoGDIs in cell lines and RBL-2H3 strains (wildtype and knockdown) were analyzed by gel electrophoresis of PCR products (RT-PCR) and quantitative PCR (qPCR) of reverse-transcribed mRNA. Briefly, total RNA was isolated using Trizol (Ambion) according to the manufacturer’s instructions. RNA concentrations were measured using a NanoVue spectrophotometer (GE Life Sciences). Complementary DNA (cDNA) was synthesized from 2 µg RNA and 0.4 µg oligo dT in a volume of 20 µl, using the SuperScript II Reverse Transcriptase kit (Invitrogen) according to manufacturer's protocol. PCR was performed using 1 µL of cDNA with 20 amplification cycles and analyzed on a 1% agarose gel stained with SYBR Safe (ThermoFisher). qPCR was performed using Mastercycler RealPlex 2 (Eppendorf) thermocycler and SensiFAST SYBR No-ROX Kit (Meridion) according to manufacturer's instructions. GAPDH was used as the internal control reference for the purpose of normalizing mRNA levels. The 2^−ΔΔCt^ values were calculated for each gene compared to the scrambled control for gene depletion experiments or compared to the empty vector control for overexpression experiments. RhoGDI mRNA levels were relevant to the scrambled control which were set to 100%. Primer sequences are listed below.

**Table:** 

Gene	Forward (5′-3′)	Reverse (5′-3′)	Product (bp)
PCR primers			
GAPDH	TGACTCTACCCACGGCAAGT	AGTGGATGCAGGGATGATGT	487
ARHGDIA	CTAGAGGTGAGCATGGCAGA	CTCCCAGGACAGGTGGTCAG	591
ARHGDIB	GCTGCAGGAGATGGACAAAG	GCAATGATCAACATGGTCCG	548
ARHGDIG	CTGGGCCTGGACGCGTGCGA	AGTCCTGGCAGACATGGAGG	664
qPCR primers			
GAPDH	ACTCCCATTTCTTCCACCTTTG	CCCTGTTGCTGTAGCCATATT	105
ARHGDIA	AATCTCTTTCCGGGTGAACAG	CGACCATGTAGTCAGTCTTGTC	101
ARHGDIB	CGTACCGGAATGGGATGAAA	CTACTGGAGTGAGGAACTCGTA	93
ARHGDIG	GTCAGTGGCCTCAAATGTCTA	TCCACTGGAGTCACAAATTCA	121
RHOA	GACCAGTTCCCAGAGGTTTATG	GTCCCATAAAGCCAACTCTACC	96
RHOC	CGTCCCTACTGTCTTTGAGAAC	CAGGCGATCGTAGTCTTCTTG	106
RHOG	CGTGCCTTCTCATCTGCTATAC	GTCCCACAGGTTTAGGTTCAC	122
RAC1	CTGTCCCAATACTCCCATCATC	GAGTCAGCTTCTTCTCCTTCAG	95
RAC2	CATGCCTTCTCATCAGCTACA	AGGTTTACTGTCCACCATCAC	101
RAC3	CCGTGATGACAAGGACACAA	GGTACTTGACAGAACCGATCTC	115
CDC42	ATGATTGGTGGAGAGCCATAC	GATGGAGAGACCACTGAGAAAC	115

### 4.4 Protein binding assays

GST-tagged Rho GTPases (RhoA, RhoG, Rac1, Rac2, and Cdc42), GST-tagged RhoGDIs and His_6_-tagged RhoGDIs (RhoGDI1 and RhoGDI2) constructs were generated in pGEX-2T and pET15b for protein production and purification. Bacterial produced GST-fusion proteins were subjected to affinity purification as described.^52^ For direct binding studies, GST-Rho GTPase proteins were immobilized on 10 µL of packed glutathione agarose beads (Sigma). Samples were incubated with 10 µg of purified His_6_-tagged RhoGDI1 or RhoGDI2 in binding buffer (1× PBS, 0.5% Triton X-100, 5 mM MgCl_2_) for 45 min to allow for binding. Beads were washed in H-buffer (20 mM HEPES, pH 7.5, 60 mM NaCl, 5 mM MgCl_2_) and analyzed by immunoblot using anti-GST (Sigma) and anti-His_5_ (Santa Cruz Biotechnology) antibodies. For endogenous Rho GTPase affinity pulldown assays, RBL-2H3 cells were grown to confluency on 10 cm plates, washed twice with PBS and lysed in lysis buffer (50 mM HEPES, pH 7.5, 50 mM NaCl, 5 mM MgCl_2_, 1% Triton X-100) with protease inhibitor cocktail (PIC: 10 μg/mL each leupeptin, pepstatin, and antipain, 0.5 μg/mL each aprotinin and bestatin, 0.5 mM each PMSF and phenanthroline). GST-tagged RhoGDI proteins were immobilized on glutathione agarose beads, then added to cell lysates and incubated for 1 h at 4 °C on a nutator. Beads were then washed with lysis buffer, eluted with elution buffer (20 mM Tris pH 6.8, 5 mM EDTA, 1% SDS, 1% Triton X-100, PIC) and analyzed by immunoblot. Samples from this pulldown were also analyzed by mass spectrometry using a nano-LC MS (Orbitrap QEactive, ThermoFisher). SEQUEST was used to generate correlation scores MS-identified peptides.

### 4.5 Subcellular fractionation and immunoblot

RBL-2H3 cells were grown to 90% confluency in 15 cm dishes and were stimulated or left unstimulated. Cells were washed with PBS (pH 7.4) and frozen in 1 mL of 10 mM HEPES pH 7.5, 0.3 M sucrose, 1 μg/mL each leupeptin and pepstatin, and 0.1 mM PMSF. Cells were thawed and scraped from the dishes and passed 10 times through a ball bearing homogenizer with a clearance of 10 microns. Lysates were centrifuged at 4,000 × *g* to generate post-nuclear homogenates. Membrane fractions were obtained by centrifugation of lysates at 20,000 × *g* and 200,000 × *g* for 1 h at 4 °C. A cytosolic fraction was taken from the supernatant of the 200,000 × *g* centrifugation. Fractions were analyzed by immunoblot for the presence of RhoGDIs using anti-RhoGDI1 antibody (Santa Cruz Biotechnology) and anti-RhoGDI2 antibody (Abcam), Rho GTPases using anti-RhoA (Santa Cruz Biotechnology), anti-RhoG (Santa Cruz Biotechnology), anti-Rac1 (EMD Millipore), anti-Rac2 (Thermo Scientific), anti-Cdc42 (Novus), ER using anti-calnexin (Cell Signaling Technology), or anti-FAM14B (Sigma) as markers, mitochondria using anti-MCU (Thermo Fisher) as a marker, and cytosol/loads using anti-β-tubulin (Abcam) as a marker. Blots were then probed with Alexa Fluor 680 goat antirabbit and Alexa Fluor 800 goat antimouse secondary antibodies (Thermo Fisher). Blots were scanned using an Odyssey CLx Infrared Imaging System (LI-COR Biosciences). Levels of Rho proteins in membrane and cytosol fractions were quantified from two blots by band densitometry calculated as the percent of homogenate after load normalization calculated using β-tubulin.

### 4.6 Immunofluorescence microscopy

Fixed-cell fluorescence microscopy was performed on RBL-2H3 cells to visualize F-actin, RhoGDI distribution, and granule distribution. Cells were either left unstimulated or stimulated with 40 ng/mL of DNP-BSA for 30 min at 37 °C. Cells were fixed for 20 min with 4% (wt/v) paraformaldehyde following stimulation. F-actin was labeled with Alexa Fluor 546 phalloidin (Thermo Fisher), RhoGDIs were labeled with anti-RhoGDI1 (Santa Cruz Biotechnology) and anti-RhoGDI2 (Abcam), granules were labeled with mouse monoclonal anti-CD63 antibodies (clone AD1, BioRad) and nuclei labeled with DAPI. Labeled secondary antibodies used were Alexa Fluor 555 donkey antimouse and Alexa Fluor 488 donkey antirabbit (Thermo Fisher). Cells were imaged using 63× objective (1.4 NA) on a Zeiss Observer Z1 epifluorescence microscope and processed with Axiovision v4.8 software.

### 4.7 Rho activation assay

Rho activation assays were performed by affinity pulldown assay as previously described.^36^ Briefly, GST-PBD (p21-binding domain which binds GTP-bound Cdc42/Rac1/Rac2), GST-RBD (Rho-binding domain which binds GTP-bound RhoA) and GST-ELMO (N-terminal RhoG binding domain of ELMO)^53^ were immobilized on 20 μL packed glutathione agarose beads (Sigma). Cells 90% confluent from a 10 cm plate were lysed in B-buffer (50 mM Tris pH 7.5, 150 mM NaCl, 0.5 mM MgCl_2_, 1% Triton X-100, PIC) and lysates were incubated with prepared beads for 30 min with nutation. Beads were washed three times in B-buffer and bound samples were analyzed by immunoblot. Levels of active Rho proteins were quantified from two blots by band densitometry calculated as percent of total.

### 4.8 Degranulation assay

Degranulation was measured by the release of β-hexosaminidase from RBL-2H3 cells. Cells were seeded in 24-well plates at a density of 150,000 cells/well and grown overnight. Following IgE sensitization and DNP-BSA stimulation, cells were placed on ice to stop degranulation. Supernatants were collected before lysing cells with 1% Triton X-100. Supernatants and cell lysates were centrifuged at 1,000 × *g* for 10 min to remove residual cells and 100 μL of cell-free supernatant or lysate was incubated with 100 μL of 1.2 mM 4-methylumbelliferyl N-acetyl-β-D-glucosaminide (MUG) (Sigma) in 0.1 M sodium citrate and 0.1 M citric acid for 30 min at 37 °C. The reaction was terminated by adding 50 μL of 0.1 M glycine (pH 10). MUG is cleaved by β-hexosaminidase to release fluorescent 4-methylumbelliferone which was measured by a Synergy-4 fluorometer (360 nm ± 20 nm excitation and 450 nm ±20 nm emission; BioTek Instruments). Fluorescence levels are proportional to degranulation, and were calculated as percentages by dividing the β-hexosaminidase activity in the supernatants divided by total β-hexosaminidase activity (determined by addition of supernatant β-hexosaminidase activity and cell lysate β-hexosaminidase activity).

### 4.9 Flow cytometry

RBL-2H3 cells were sensitized with anti-DNP-IgE for 3.5 h and stimulated with DNP-BSA for 30 min or left unstimulated. Cells were then fixed with 4% (wt/v) paraformaldehyde and suspended in flow cytometry buffer (PBS, 1 g/L BSA). Cells were stained with mouse anti-FcεRIα antibody (EMD Millipore) and Alexa Fluor 555 donkey antimouse antibody (Thermo Fisher). A BD LSRFortessa Cell Analyzer was used to detect cell surface fluorescence and BD FACSDiva software was used for data analysis. The median fluorescence intensity was used as a measure of FcεRI levels.

### 4.10 Statistical analysis

Graphs show the statistical means, ± SD of the means with samples size (n) as indicated. Comparative analyses were performed by Student's *t*-test with Bonferroni correction when performing multiple comparisons.

## Supplementary Material

qiae150_Supplementary_Data
