# Supplementary material for: RhoGDI in RBL-2H3 cells acts as a negative regulator of Rho GTPase signaling to inhibit granule exocytosis
Source: J Leukoc Biol. 2024 Jun 29;116(6):1498–514. doi: 10.1093/jleuko/qiae150 (PMC11599123; doi:10.1093/jleuko/qiae150)
Supplement: qiae150_Supplementary_Data [file qiae150_supplementary_data.pdf]

Supplemental Data

Zhang et al., 2024

RhoGDI in RBL-2H3 cells acts as a negative regulator of Rho GTPase signaling to inhibit granule exocytosis

Eric L. Zhang, Jennifer Van Petten and Gary Eitzen
Department of Cell Biology, University of Alberta, Edmonton, Alberta, Canada

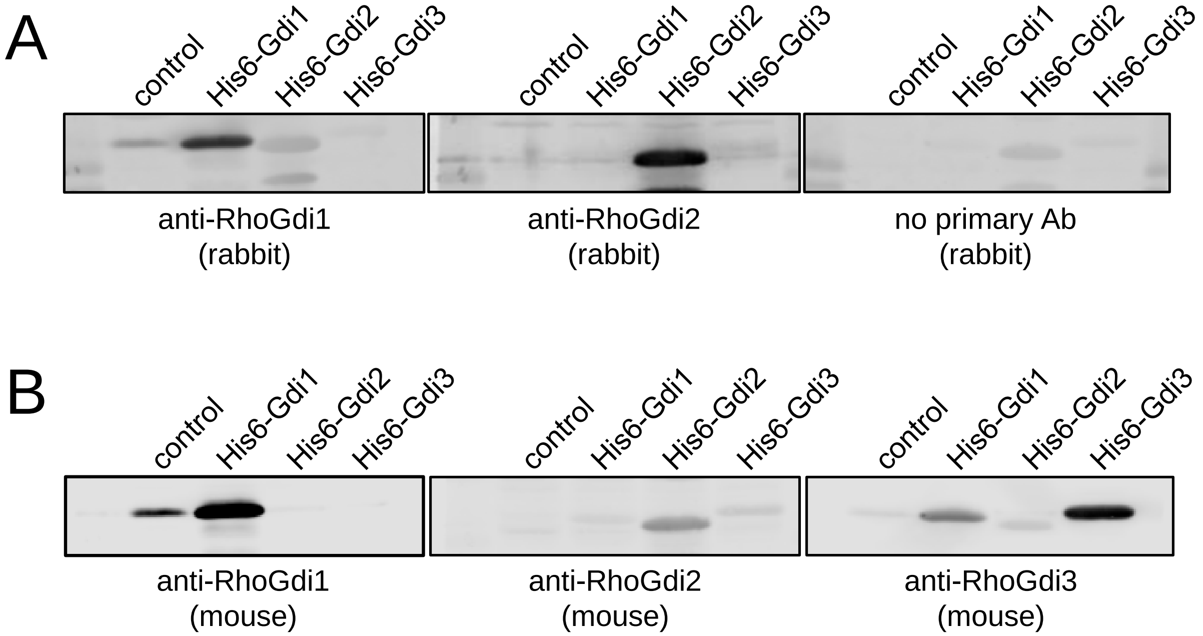

Figure S1. RhoGDI antibody test. Full-length His_6-RhoGDI1 and His_6-RhoGDI2, and N-terminal truncated (Δ27 a.a.) His_6-RhoGDI3 were expressed in E. coli, purified and 0.1 μg analyzed by SDS-PAGE and immunoblot with the indicated antibody. Control indicates RBL-2H3 lysate. Blot were scanned on a Odyssey CLx imaging system (LI-COR). RhoGDI antibodies against RhoGDI1, RhoGDI2 and RhoGDI3 were sources from:

Rabbit anti-RhoGdi1 (RhoGDIα), 0.20 μg/ml, SCBT A-20
Rabbit anti-RhoGdi2 (D4-GDI), 0.36 μg/ml, Abcam EPR14212(B)
Mouse anti-RhoGdi1 (RhoGDIα), 0.12 μg/ml, SCBT G-2
Mouse anti-RhoGdi2 (Ly-GDI), 0.20 μg/ml, SCBT E-7
Mouse anti-RhoGdi3 (RhoGDIγ), 0.20 μg/ml, SCBT E-1

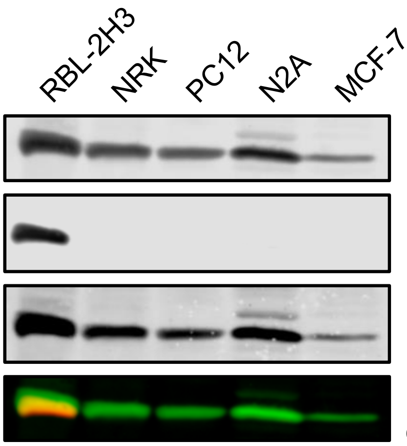

Figure S2. Expression RhoGDI isoforms in tissue culture cell lines. 20 μg of lysates from the indicated cell lines were analyzed by SDS-PAGE and immunoblot. Blot were scanned on a LiCor Odyssey imaging system. *anti-RhoGdi3 antibodies cross-react with RhoGdi1 (see Figure S1, supplemental data).

- Mouse anti-RhoGdi1 (RhoGDIα), 0.12 μg/ml, SCBT G-2
- Rabbit anti-RhoGdi2 (D4-GDI), 0.36 μg/ml, Abcam EPR14212(B)
- Mouse anti-RhoGdi3 (RhoGDIγ), 0.20 μg/ml, SCBT E-1

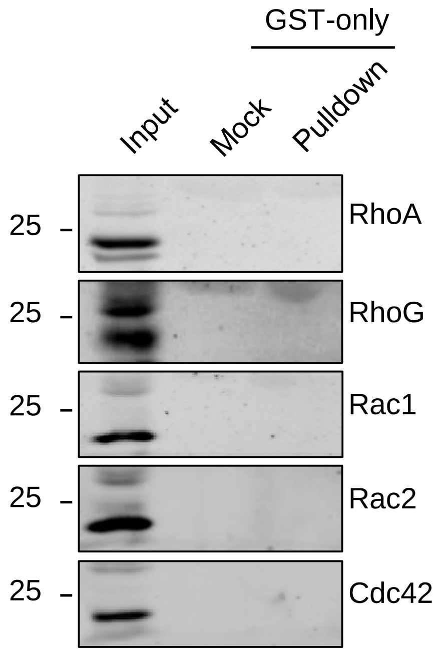

Figure S3. GST-only control for GST-RhoGDI affinity pulldown analysis of Rho GTPases. RBL-2H3 cell lysates were incubated with GST immobilized on glutathione beads. Affinity pulldown samples were separated by SDS-PAGE and analyzed by immunoblotting. Lanes labeled *Mock* indicated beads incubated with lysis buffer instead of lysate. Immunoblots were probed with antibodies against RhoA, RhoG, Rac1, Rac2, and Cdc42. The control GST-only pulldown shows no background binding of Rho proteins to GST or the glutathione resin.

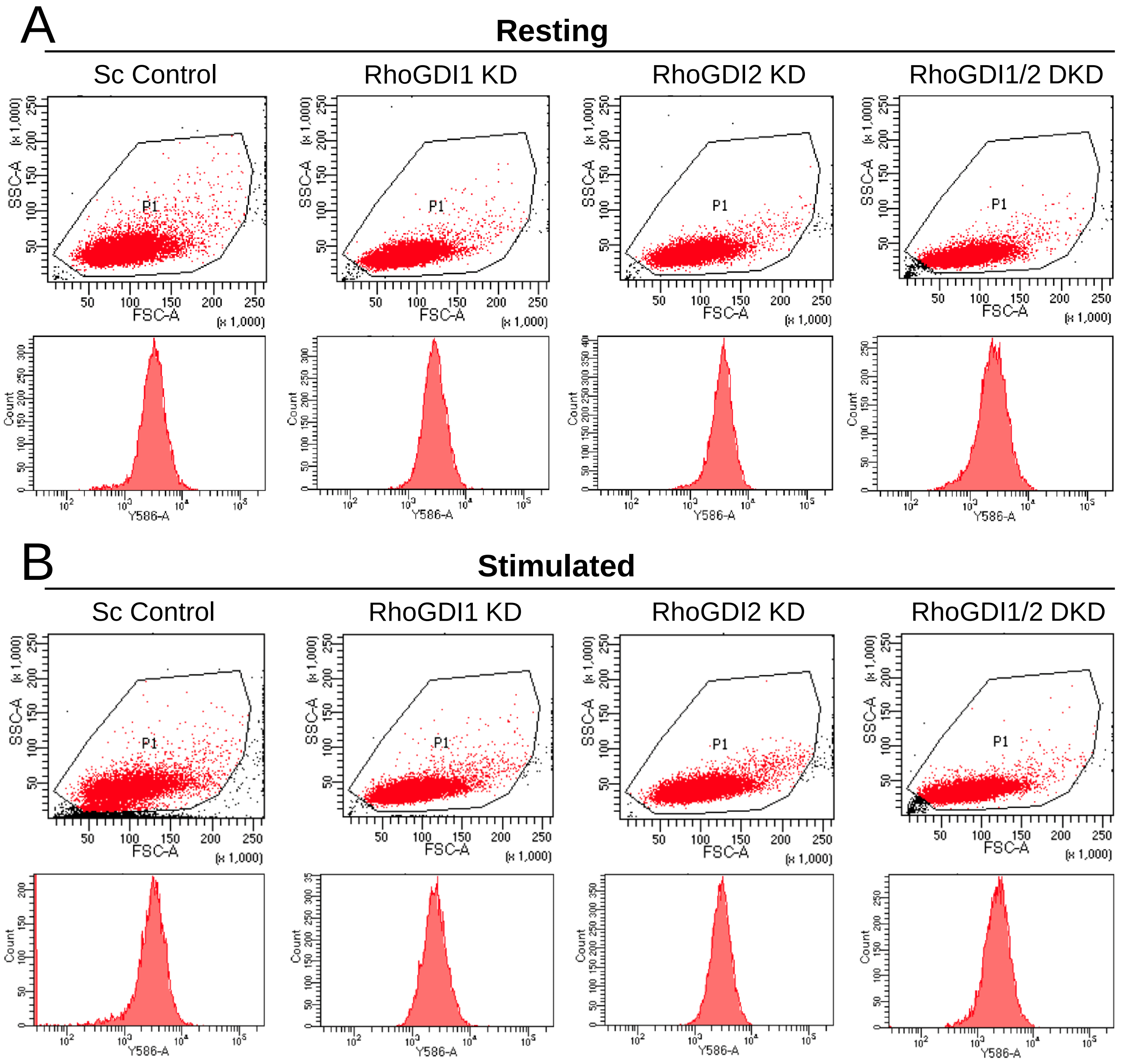

C

Resting	Y586-A Median				Stimulated	Y586-A Median			
	1	2	3	4		1	2	3	4
Sc Control	2986	3917	2534	2020	Sc Control	2823	3183	3280	1966
RhoGDI1 KD	2675	3098	2371	1732	RhoGDI1 KD	2304	2426	2216	2070
RhoGDI2 KD	3380	3989	3058	2497	RhoGDI2 KD	2724	2911	2814	2202
RhoGDI1/2 DKD	2305	2440	2996	2353	RhoGDI1/2 DKD	2140	1923	2042	1816

Figure S4. Example dot plots and histograms for flow cytometry analysis of FcεRI surface expression. RBL-2H3 cells were either left resting **(A)** or antigen-stimulated for 30 minutes **(B)**, then fixed and labelled with anti-rat FcεRI antibodies without permeabilization to label surface exposed receptors. *Upper panels,* dot plots of forward and side scatter. *Lower panels*, fluorescence levels of cells gated in P1. **(C)** Median fluorescence from four biological replicates.
